# Safety of early pelvic drain removal in colorectal surgery based on drainage quantity

**DOI:** 10.1186/s12893-023-02041-3

**Published:** 2023-05-16

**Authors:** Kosuke Yoshimura, Hiroki Ohge, Yusuke Watadani, Shinnosuke Uegami, Ikki Nakashima, Toshinori Hirano, Kensuke Shimbara, Hirofumi Doi, Shinya Takahashi

**Affiliations:** Department of Surgery, Graduate School of Biomedical and Health Sciences, Hiroshima University, 1-2-3 Kasumi, Minami-Ku, Hiroshima City, Hiroshima Prefecture 734-8551 Japan

**Keywords:** Pelvic drain, Drainage quantity, Colorectal surgery, Postoperative complication

## Abstract

**Background:**

This study aimed to investigate the association between the drainage quantity of pelvic drains and postoperative complications in colorectal surgery.

**Materials and methods:**

This retrospective single-center study enrolled 122 colorectal surgery patients between January 2017 and December 2020. After restorative proctectomy or proctocolectomy with gastrointestinal anastomosis, a continuous, low-pressure suction pelvic drain was placed and its contents measured. Removal ensued following the absence of turbidity and a drainage quantity of ≤ 150 mL/day.

**Results:**

Seventy-five patients (61.5%) and 47 patients (38.5%) underwent restorative proctectomy and proctocolectomy, respectively. Drainage quantity changes were observed on postoperative day (POD) 3, regardless of the surgical procedure or postoperative complications. The median (interquartile range) number of PODs before drain removal and organ-space surgical site infection (SSI) diagnosis were 3 (3‒5) and 7 (5‒8), respectively. Twenty-one patients developed organ-space SSIs. Drains were left in place in two patients after POD 3 owing to large drainage quantities. Drainage quality changes enabled diagnosis in two patients (1.6%). Four patients responded to therapeutic drains (3.3%).

**Conclusions:**

The drainage quantity of negative-pressure closed suction drains diminishes shortly after surgery, regardless of the postoperative course. It is not an effective diagnostic or therapeutic drain for organ-space SSI. This supports early drain removal based on drainage quantity changes in actual clinical practice.

**Trial registration:**

The study protocol was retrospectively registered and carried out per the Declaration of Helsinki and approved by the Hiroshima University Institutional Review Board (approval number: E-2559).

## Background

Total mesorectal excision (TME) and tumor-specific mesorectal excision (TSME) are important procedures in colorectal surgery. Crucial treatment strategies aimed at sphincter preservation are currently being implemented even if the tumors are located in the lower anorectum. Several opportunities for anastomosis are now accessible, not only on the distal side of the peritoneal reflection but also in the anal canal or anus. However, anastomosis within 5 cm of the proximal side of the anal verge increases the risk of suture failure by 5.4–6.5 times compared with anastomosis > 5 cm away from the proximal side of the anal verge [1, 2].

Consequently, gastrointestinal suture failure, chyle leakage, or residual hematoma, conditions caused by intraoperative pelvic manipulation, are common causes of postoperative intra-abdominal infection, specifically, organ-space surgical site infection (SSI). Organ-space SSI can delay wound healing, increase retreatment rates, increase postoperative mortality, and greatly affect long-term anorectal functions. Pelvic drains are often used during colorectal surgery to prevent and detect organ-space SSI and determine treatment. However, there are no scientific justifications for using pelvic drains to protect from organ-space SSI caused by colectomy suture failure [3], and there is a trend against their placement in recent years. In cases of rectal resection with TME or TSME, some clinicians consider a pelvic drain as an effective measure against organ-space SSI due to suture failure [4], whereas others believe it has little effect [5, 6]. Most studies defined the primary endpoint of placing surgical drains as the presence or absence of postoperative organ-space SSI. However, these studies may be limited by the different types of drains used. To our knowledge, few studies have examined the association between pelvic drains and postoperative complications, mainly organ-space SSI, by focusing on drainage quantity and properties in clinical practice.

In this study, we investigated the relationship between drainage quantity and properties of pelvic drain and postoperative complications. Based on the results, we examined the significance of placing a pelvic drain after gastrointestinal anastomosis following proctectomy or proctocolectomy with TME or TSME.

## Methods

### Study design and patients

This retrospective single-center study included patients who underwent elective surgery for colorectal diseases and pelvic drain placement during surgery. Surgery was performed between January 2017 and December 2020 at Hiroshima University Hospital. Colorectal diseases included sporadic rectal cancer and inflammatory bowel disease (ulcerative colitis [UC] or ulcerative colitis-associated cancer [UCAC], Crohn’s disease, and diverticulitis). In cases of UC or UCAC, we performed restorative total proctocolectomy. Restorative proctectomy was performed for other diseases. Patients with uncontrolled intraperitoneal abscesses due to internal fistula formation or intestinal perforation before surgery were excluded from this study. The study protocol was carried out per the Declaration of Helsinki and approved by the Hiroshima University Institutional Review Board (approval number: E-2559). Informed consent was obtained from all participates.

### Perioperative management

Mechanical bowel preparation was performed preoperatively. One sachet of sodium–potassium-ascorbic acid combination powder (244.212 g) was dissolved in 2000 mL of water and administered orally in the afternoon of the day before surgery. One bottle of sodium picosulfate hydrate (10 mL) was taken orally before sleep, and glycerin was administered as an enema on the morning of surgery. Prophylactic antibacterial agents included cefmetazole sodium. Mechanical bowel preparation and prophylactic antimicrobial administration were consistent throughout the observation period.

After proctectomy or proctocolectomy, gastrointestinal anastomosis was performed. For proctectomy, we performed a double stapling technique using PROXIMATE® ILS Curved Intraluminal Stapler (CDH25A or CDH29A, Johnson & Johnson Co., Tokyo, Japan). In contrast, proctocolectomy was performed with the hand-sewing (ileal pouch-anal anastomosis) technique. After irrigating the pelvis with physiological saline solution and suctioning the accumulated fluid, a 19-Fr. Blake silicon drain® (Johnson & Johnson Co.) was inserted and positioned on the pelvic floor (anterior to the sacral bone) percutaneously. After abdominal closure, the drain was connected to a J-VAC® drainage system (standard type, Johnson & Johnson Co.), which provided a continuous low-pressure suction via the rebound of an internal spring to create a closed drain. Abdominal X-ray was performed immediately after surgery in all cases to ensure that the tip of the drain was positioned properly on the pelvic floor. We confirmed the position of the tip of drain by routine abdominal X-ray performed from the postoperative day (POD) 1. The daily drainage quantity was calculated at 9:00 am. The following criteria for drain removal were based on a previous study by our department [7]: absence of turbidity (from chyle leakage and secondary hemorrhage) and drainage quantity ≤ 150 mL/day. Organ-space SSI was defined as an abdominal abscess due to extraintestinal leakage or fluid accumulation within 30 days postoperatively. Extraintestinal leakage and fluid accumulation were confirmed by contrast-enhanced computed tomography (CT) images or barium enema. Pathogens were isolated from either the drainage fluid or aspirated fluid in all cases of organ-space SSI.

### Data collection and statistical analysis

A review of all the patients’ medical records allowed us to obtain data on preoperative diagnoses, the American Society of Anesthesiologists (ASA) preoperative statuses, blood test results, surgical procedures, construction of diverting loop ileostomy, operation times, intraoperative bleeding, and use of blood transfusions. The primary outcomes were drainage quantity and properties from the day of surgery. The secondary outcomes were postoperative complications and the date of drain removal.

The JMP® Pro 15.0.0 software (SAS Institute Inc., Cary, NC) was used for statistical analysis. Continuous variables are expressed as mean (± standard deviation [SD]) or median (interquartile range [IQR]). Paired or independent groups were compared using the Wilcoxon signed-rank test, Mann–Whitney U test, or Fisher’s exact test as applicable. Statistical significance was set at *P* < 0.05.

### Ethical considerations

Participants were informed about the study, including its objectives, and could terminate their participation whenever they wished. As the opt-out method was used, this information was provided online without requiring informed consent from patients.

## Results

From January 2017 to December 2020, a total of 122 patients underwent restorative proctectomy or proctocolectomy in our department. Of the 122 included patients, 83 were men (68.0%), the median (IQR) age at surgery was 62.5 (51‒70) years, and the median (IQR) preoperative body mass index was 22.2 (20.2‒24.6) kg/m^2^. The preoperative diagnosis was sporadic rectal cancer in 70 patients (57.4%) and inflammatory bowel disease in 52 patients (42.6%). Blood tests showed mean (± SD) hemoglobin and median (IQR) albumin levels of 12.7 (± 1.8) g/dL and 4.0 (3.6‒4.4) g/dL, respectively. Most cases had an ASA-performance status class 2 (105 cases, 86.1%). Detailed patients’ characteristics are shown in Table 1.

**Table 1:** Preoperative characteristics of the 122 included patients

	*n* = 122
Sex: male, n (%)	83 (68.0)
Age, median (IQR), years	62.5 (51–70)
BMI, median (IQR), kg/m^2^	22.2 (20.2–24.6)
Preoperative diagnosis	
Sporadic rectal cancer, n (%)	70 (57.4)
IBD, n (%)	52 (42.6)
Blood test results	
Hemoglobin level, mean (± SD), g/dL	12.7 (± 1.78)
Albumin level, median (IQR), g/dL	4.0 (3.6–4.4)
ASA-PS, 1/2/3, n (%)	8 (6.6)/105 (86.1)/9 (7.4)

*IQR* Interquartile range, *BMI* Body mass index, *IBD* Inflammatory bowel disease, *SD* Standard deviation, *ASA-PS* American society of anesthesiologists-performance status

The perioperative results are presented in Table 2. Restorative proctectomy was performed in 75 (61.5%) patients (high anterior resections, 56 cases; low anterior resections, 19 cases) while restorative proctocolectomy was performed in 47 (38.5%) patients. A diverting loop ileostomy was created in 62 patients (50.8%). The median (IQR) volume of intraoperative bleeding was 103 mL (24 − 379). Three patients (2.5%) required intraoperative blood transfusions. The median (IQR) drain removal day was POD 3 (3 − 5). The drains were left in place after POD 3 in 16 patients (13.1%) because the drainage quantity was ≥ 150 mL/day without turbidity. Concerning postoperative complications, organ-space SSIs occurred in 21 patients (17.2%) due to pelvic abscesses caused by suture failure in 12 cases and postoperative residual abscesses without suture failure in nine cases. None of these were caused by the placed drains (e.g. suture failure due to mechanical stimulation of the anastomotic site or retrograde infection). Seven cases of organ-space SSI were diagnosed while pelvic drains were in place. Of these, organ-space SSIs occurred in two of the 16 patients in whom drains were left in place after POD 3 owing to large drainage quantities.

**Table 2:** Surgical procedure and postoperative results

	*n* = 122
Surgical procedure	
Restorative proctectomy, n (%)	75(61.5)
Restorative proctocolectomy, n (%)	47 (38.5)
Diverting loop ileostomy, n (%)	62 (50.8)
Bleeding volume, median (IQR), mL	103 (24–379)
Intraoperative transfusion, n (%)	3 (2.5)
Drain removal day, median (IQR), days	3 (3–5)
Organ-space SSI	
Pelvic abscess due to suture failure, n (%)	12 (9.8)
Residual abscess without suture failure, n (%)	9 (7.4)

*IQR* Interquartile range, *SSI* Surgical site infection

**Fig. 1:**
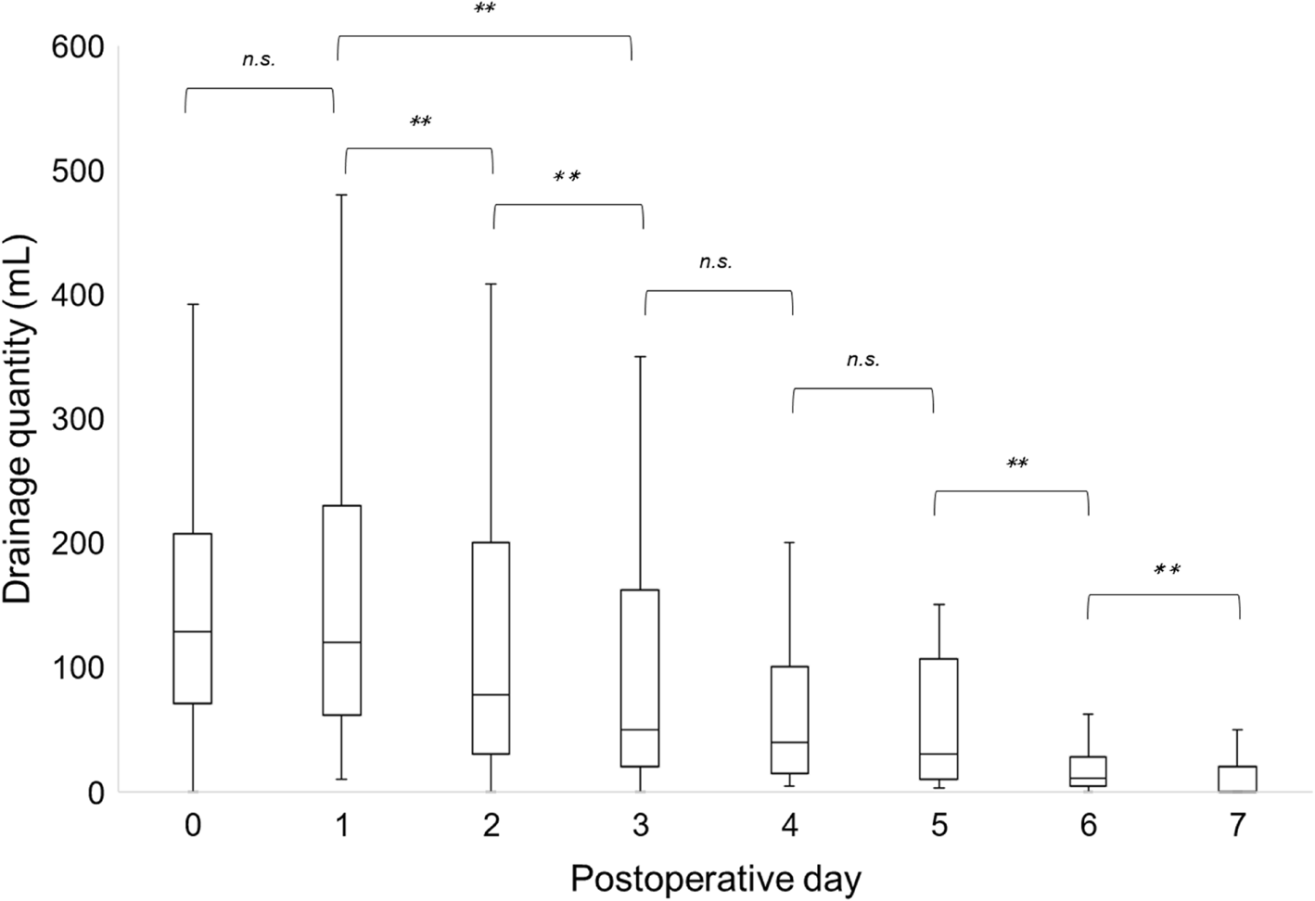
Changes in median daily drainage quantity in all 122 cases. The daily drainage quantity decreased significantly up to POD 3. No significant differences were observed thereafter. POD, postoperative day. **P < 0.01, n.s., not significant

**Fig. 2:**
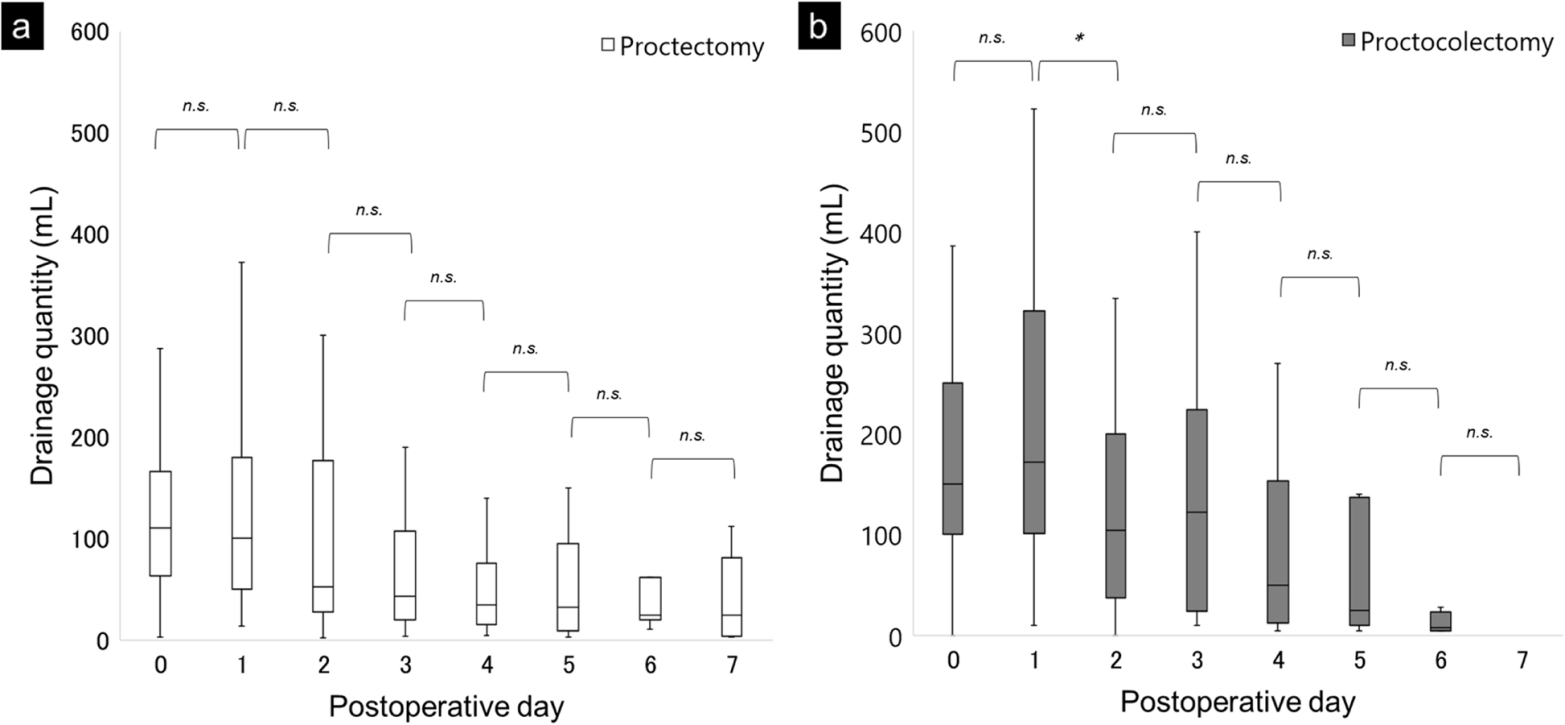
Changes in median daily drainage quantity by surgical procedure. No significant change was observed for proctectomy (**a**), while the drainage quantity decreased significantly from POD 1 to 2 in patients who underwent proctocolectomy (**b**). POD, postoperative day. *P < 0.05, n.s., not significant

**Fig. 3:**
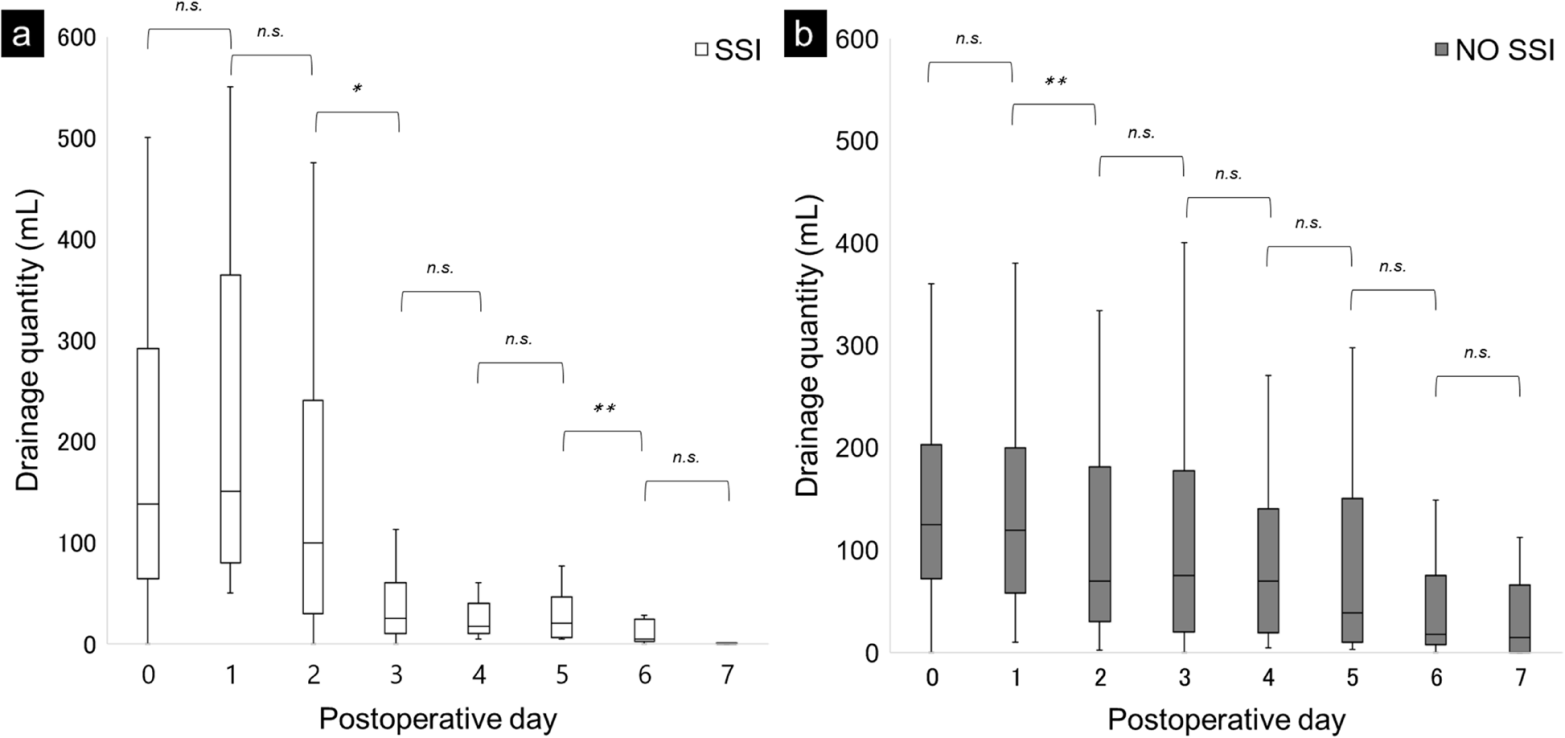
Changes in median daily drainage quantity by the presence of SSI. The drainage quantity decreased significantly from POD 2 to 3 in the SSI group (**a**) and from POD 1 to 2 in the non-SSI group (**b**). POD, postoperative day; SSI, surgical site infection. *P < 0.05, **P < 0.01, n.s., not significant

**Fig. 4:**
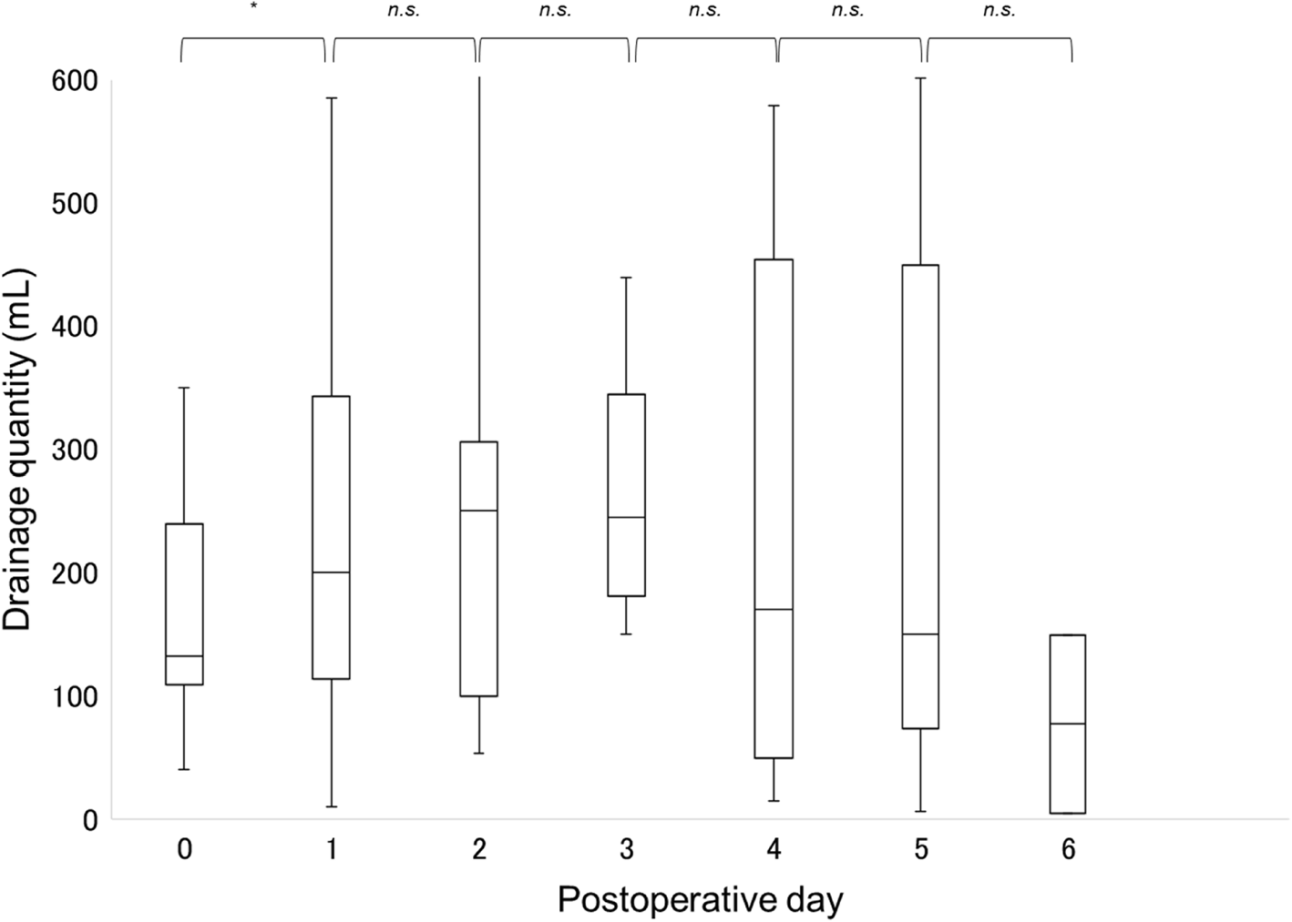
Changes in median daily drainage quantity in 16 cases with drainage quantity greater than 150 mL/day on POD 3. The daily drainage quantity changed significantly up to POD 1. No significant differences were observed thereafter. POD, postoperative day. *P < 0.05, n.s., not significant

In all 122 patients, the daily drainage quantity decreased significantly from POD 1 to 3 (Fig. 1). None of the cases of postoperative secondary hemorrhage required hemostatic interventions. A comparison of daily drainage quantity by surgical procedures showed that the volume decreased significantly from POD 1 to 2 for proctocolectomy, while no significant change was observed for proctectomy (Fig. 2). Similar analysis showed that the drainage quantity decreased significantly from up to POD 3 in patients with diverting loop ileostomy, while no significant change was observed in patients without diverting loop ileostomy. A correlation analysis between the daily drainage quantity and the presence of organ-space SSI showed that the volume decreased significantly from POD 2 to 3 for the SSI group, whereas it decreased significantly from POD 1 to 2 for the non-SSI group (Fig. 3). In addition, no significant difference was shown in the incidence of SSI with or without ileostomy (24.2% vs 10.0%, *P* = 0.054). There were significant changes in drainage quantity from POD 0 to 1 in cases with ≥ 150 mL/day drainage volume in POD 3 (Fig. 4). The details of the 21 cases of organ-space SSIs are summarized in Table 3. The median (IQR) POD of organ-space SSI diagnosis was 7 (5‒8). All cases were diagnosed using radiological examinations, such as CT or barium enema. Prior to diagnosis, changes in the properties of the drainage fluid occurred only in two cases. Physical symptoms, such as fever and abdominal pain, occurred in 12 patients, and an increased inflammatory response, as shown by blood test results, occurred in nine patients. The treatments involved converting the drain placed intraoperatively to a therapeutic drain (four cases), administration of antibiotics (six cases), placement of a new drain via interventional radiology (IVR) (eight cases), and reoperation (three cases). The reoperations included stoma construction in two cases and intestinal resection with re-anastomosis in one case.

**Table 3:** Clinical details of organ-space SSI cases

	*n* = 21
With diverting loop ileostomy, n (%)	15 (71.4)
Day of diagnosis, median (IQR), day	7 (5–8)
Diagnosis opportunity (multiple allowed)	
Physical symptoms, n (%)	12 (57.1)
Blood test results, n (%)	9 (42.9)
Change in drainage properties, n (%)	2 (9.5)
Isolation of microbial pathogens	
*Escherichia coli*, n (%)	4 (19.0)
*Enterococcus faecalis*, n (%)	4 (19.0)
*Enterobacter cloacae*, n (%)	2 (9.5)
Others	11(52.5)
Treatment	
Conversion to therapeutic drain, n (%)	4 (19.0)
Antibiotics only, n (%)	6 (28.6)
New drain placement with IVR, n (%)	8 (38.1)
Reoperation, n (%)	3 (14.3)

*SSI* Surgical site infection, *IQR* Interquartile range, *IVR* Interventional radiology

## Discussion

In this study, we investigated the association of drainage quantity and properties of pelvic drains with postoperative complications. There was a significant decrease in the daily drainage quantity at POD 3, regardless of the surgical procedure or the presence of organ-space SSI. In addition, changes in drainage properties did not provide many opportunities to diagnose organ-space SSI (2/122 cases, 1.6%). Although, the drainage quantity remained high over a long period in a few cases, it was not associated with organ-space SSI onset. These results indicate that the diagnosis of early postoperative complications (mainly suture failure) through drainage quantity is valid, but not necessarily valid for the diagnosis of late postoperative complications, in which drainage quantity is reduced. Even when the drainage quantity did not decrease, the same results were observed in this study. Few studies have verified the functions of pelvic drains by focusing on the drainage quantity and properties in clinical practice. Also, there is no consensus on whether drain placement in proctectomy reduces the incidence of organ-space SSI. To our knowledge, this is the first study to examine the occurrence of postoperative complications, mainly organ-space SSI, by focusing on the daily drainage quantity.

According to US guidelines, the purpose of drain placement includes early detection of postoperative secondary hemorrhage and removal of blood and body fluids accumulating from surgical manipulations [8]. Some reports support the need for blood and body fluids removal. Furukawa et al. examined bacterial cultures of ascites in colorectal cancer surgery [9]. They collected samples near the intestinal anastomosis immediately before closing the abdomen. This study reported that 42 of 49 cases (85.7%) were positive for intra-abdominal bacterial contamination, and 36.4% were positive for *Bacteroides fragilis*. Moreover, proctectomy results in extensive loss of the pelvic peritoneum and reduced capacity to absorb body fluids [10]. Therefore, in postoperative management, it is important to remove the accumulated fluid using pelvic drains placed during surgery. In this study, there was a significantly high daily drainage quantity in the acute postoperative period. These findings suggest that postoperative acute drainage by pelvic drain is a promising method for removing body fluids that can otherwise serve as a growth medium for bacteria.

Recently, organ-space SSI was observed in 17.1% of patients who underwent proctectomy to preserve the anus [6, 11]. Some researchers consider intraoperative drain placement useful, as suggested by one report wherein patients with drains had significantly fewer reoperations [12] and another report wherein postoperative mortality was 30% lower in the drain group than in the non-drain group [13]. However, unlike in colectomy, there is no consensus on whether drain placement in restorative proctectomy reduces the incidence of organ-space SSI. Moreover, few studies have verified the functions of specific pelvic drains by focusing on the drainage quantity and properties in clinical practice. We observed that there was a significant decrease in the daily drainage quantity at POD 3, regardless of the surgical procedure (included ileostomy construction) or the presence of organ-space SSI. On the other hand, organ-space SSIs occurred in 21 patients (17.2%); the incidence was equal to or lower than that previously reported [6, 12, 13]. These results suggested that it was difficult to correlate the drainage quantity with the occurrence of organ-space SSI. Diagnosing organ-space SSI based on drainage status was also infrequent. In this study, we did not observe any drain-related complications. But Tsujinaka et al. [4] reported that pelvic drain placement led to complications in eight of 196 patients (4.1%) who underwent anterior resection for rectal cancer, including six cases of organ-space SSIs (five retrograde infections via the drain, and one case where the drain tip mistakenly entered the intestinal anastomosis). This serves as an indication that drain-related complications should not be underestimated.

It is important to understand what potential role pelvic drains may play when organ-space SSI occurs. In the current study, more than half of the organ-space SSI cases that occurred were diagnosed after the drains were removed, when the drainage effect declined. Even when a drain was still in place, the drain tip was away from the abscess cavity. Thus, effective abscess drainage could not be expected. In this study, CT examination revealed that the drain tip was far from the abscess cavity in three of the seven cases, and effective drainage was not expected, i.e., it was difficult to convert to a therapeutic drain.

This study has several limitations. First, it was a single-center, retrospective study with a small sample size. Second, the duration of surgery was not uniform; hence, the observation times for POD 0 varied. This made accurate comparisons of drainage quantity on the succeeding days more difficult. Third, the drain placement itself may affect the amount of body fluid in the pelvis and the occurrence of organ-space SSI. However, no previous studies reported the body fluid measurement without drain placement. Also, no previous studies examined the association between the body fluid measurement and organ-space SSI. According to this retrospective nature study, it was difficult to assess these points. But for early detection of postoperative secondary hemorrhage and removal of postoperative blood and body fluids, which may be contaminated with intra-abdominal bacteria [9], it is worthwhile to place the pelvic drain until drainage quantity decreases. Finally, if the tip of the drain was placed in the abscess cavity where the organ-space SSI occurred, the attending surgeon opted for reoperation due to the patient’s general condition.

## Conclusion

The drainage quantity of negative-pressure closed suction drains placed during colorectal surgery decreased rapidly postoperatively and was not associated with the surgical procedure or the postoperative course. Changes in drainage properties may not help diagnose organ-space SSIs. Moreover, the pelvic drain could be converted to a therapeutic drain in only a limited number of cases. These findings support early drain removal based on the transition of drainage quantity in clinical practice.

## Data Availability

The authors declare that the data supporting the findings of this study are available in this article.

## Abbreviations

TME: Total mesorectal excision
TSME: Tumor-specific mesorectal excision
SSI: Surgical site infection
SD: Standard deviation
IQR: Interquartile range
POD: Postoperative day
IVR: Interventional radiology
